# Distinct Expression and Clinical Significance of Zinc Finger AN-1-Type Containing 4 in Oral Squamous Cell Carcinomas

**DOI:** 10.3390/jcm7120534

**Published:** 2018-12-10

**Authors:** Julián Suarez-Canto, Faustino Julián Suárez-Sánchez, Francisco Domínguez-Iglesias, Gonzalo Hernández-Vallejo, Juana M. García-Pedrero, Juan C. de Vicente

**Affiliations:** 1 395 Los Prados Cabueñes, 33394 Gijón, Asturias, Spain; juliansuarezcanto@gmail.com; 2 Department of Pathology, Hospital Universitario de Cabueñes, 395 Los Prados Cabueñes, 33394 Gijón, Asturias, Spain; faustinosuarezsanchez@gmail.com (F.J.S.S.); fdoig59@yahoo.es (F.D.I.); 3 School of Dentristry, Complutense University of Madrid, Pza. Ramón y Cajal, s/n, Ciudad Universitaria, 28040 Madrid, Spain; ghervall@odon.ucm.es; 4 Department of Otolaryngology, Hospital Universitario Central de Asturias (HUCA), C/Carretera de Rubín, s/n, 33011 Oviedo, Asturias, Spain; 5 Instituto de Investigación Sanitaria del Principado de Asturias (ISPA), Instituto Universitario de Oncología del Principado de Asturias (IUOPA), Universidad de Oviedo, C/Carretera de Rubín, s/n, 33011 Oviedo, Asturias, Spain; 6 Ciber de Cáncer (CIBERONC), Instituto de Salud Carlos III, Av. Monforte de Lemos, 3-5, 28029 Madrid, Spain; 7 Department of Oral and Maxillofacial Surgery, Hospital Universitario Central de Asturias (HUCA), C/Carretera de Rubín, s/n, 33011 Oviedo, Asturias, Spain; 8 Department of Surgery, University of Oviedo, Av. Julián Clavería, 6, 33006 Oviedo, Asturias, Spain

**Keywords:** ZFAND4, ANUBL1, oral squamous cell carcinoma, immunohistochemistry, prognosis

## Abstract

Zinc finger AN1-type containing 4 (ZFAND4) has emerged as a promising prognostic marker and predictor of metastasis for patients with oral squamous cell carcinoma (OSCC). However, further validation is fundamental before clinical implementation. Hence, this study evaluated the expression pattern of ZFAND4 protein expression by immunohistochemistry using an independent cohort of 125 patients with OSCC, and correlations with the clinicopathologic parameters and disease outcome. Remarkably, ZFAND4 expression, while negligible in normal epithelium, exhibited two distinct expression patterns in tumors that did not overlap. A gross granular staining was characteristic of the undifferentiated cells at the invasive front of tumors, whereas the most differentiated cells located at the center of the tumor nests showed diffuse non-granular staining. ZFAND4 staining was higher in undifferentiated than in differentiated areas of tumors. High ZFAND4 expression in differentiated cells was significantly associated to well-differentiated (*p* = 0.04) and non-recurrent tumors (*p* = 0.04), whereas ZFAND4 expression in undifferentiated cells correlated with tumor location (*p* = 0.005). No correlations between the ZFAND4 expression and patient survival were found. These data question the clinical relevance of ZFAND4 expression as a prognostic biomarker in OSCC, and also reveal distinct ZFAND4 expression patterns depending on the differentiation areas of tumors that should be evaluated separately.

## 1. Introduction

Head and neck squamous cell carcinoma that includes, among others, oral squamous cell carcinoma (OSCC) is the sixth most common cancer in the world, with an annual prevalence of nearly 600,000 new cases worldwide [1,2]. It is generally accepted that OSCC initiates and progresses through a series of multiple genetic alterations caused by chronic exposure to carcinogens, such as alcohol, smoking, and human papilloma virus [3]. Multiple genetic and molecular studies have improved our understanding of the molecular basis of this disease. Indeed, several cellular signaling pathways have been found dysregulated in these tumors through genetic and epigenetic alterations. However, despite major advances in diagnosis and treatment, the survival rate of patients with OSCC has modestly improved over the past 40 years, and it remains at approximately 50% [4].

Sasahira et al. [5] investigated the transcriptional profiles of primary and recurrent OSCC, and found that one of the most upregulated genes identified in recurrent OSCC was zinc finger AN1-type containing 4 (ZFAND4), also known as AN1 and ubiquitin-like homolog (ANUBL1). Although the functional role of ZFAND4 in cancer is still unknown, Kurihara-Shimomura et al. [6] evaluated its prognostic utility in OSCC. They concluded that ZFAND4 could be a useful marker for predicting metastasis and poor prognosis in patients with OSCC. In addition, Tang et al. [7] demonstrated that ZFAND4 expression is upregulated in gastric cancer and positively associated with the grading of this disease.

In the light of these data, ZFAND4 emerges as a promising prognostic biomarker; however, further validation in independent study cohorts is fundamental for implementation to the clinic. Therefore, the objective of this study was to investigate the expression pattern and clinical relevance of ZFAND4 protein expression using an independent cohort of 125 patients with OSCC, and to establish correlations with the clinicopathologic parameters and disease outcome.

## 2. Experimental Section

### 2.1. Patients and Tissue Specimens

A retrospective study was designed. Surgical tissue specimens from 125 patients with OSCC who underwent surgical treatment with curative purposes at the Hospital Universitario Central de Asturias between 1996 and 2007 were retrospectively collected, in accordance to approved institutional review board guidelines. All experimental procedures were conducted in accordance to the Declaration of Helsinki and approved by the Institutional Ethics Committee of the Hospital Universitario Central de Asturias and by the Regional CEIC from Principado de Asturias. Informed consent was obtained from all patients. Clinicopathologic data were collected from medical records. Tissue specimens were obtained from the Biobanco del Principado de Asturias, and representative tissue sections from archival, formalin-fixed paraffin-embedded blocks.

### 2.2. Tissue Microarray Construction

The original archived hematoxylin- and eosin-stained slides were reviewed by an experienced pathologist (FDI) to confirm histological diagnosis. Three representative tissue cores (1 mm diameter) were selected from each tumor block, and transferred to a recipient ‘Master’ block in a grid-like manner using a manual tissue microarray instrument. In addition, each tissue microarray also contained three cores of normal epithelium as an internal control. A section from each microarray was stained with hematoxylin and eosin, and examined by light microscopy to check the adequacy of tissue sampling.

### 2.3. Immunohistochemistry (IHC)

TMA sections (4 μm) were cut and dried and dried on Flex IHC microscope slides (Dako). The sections were deparaffinized with standard xylene, hydrated through graded alcohols into water, and pretreated by hydrogen peroxide to quench the endogenous peroxidase activity. Antigen retrieval was performed using Envision Flex Target Retrieval solution (Dako), at room temperature on an automatic staining workstation (DakoAutostainer Plus, Dako, Glostrup, Denmark). Staining was carried out at room temperature on an automatic staining workstation (Dako Autostainer Plus) with anti-ZFAND4 antibody (Atlas Antibodies, Stockholm, Sweden) diluted to 0.5 Nµg/mL using the Dako EnVision detection system (Dako, Glostrup, Denmark). Sections were counterstained with hematoxylin, dehydrated with ethanol, and permanently coverslipped. For negative control purposes, DakoCytomation mouse serum diluted at the same concentration as the primary antibody was used.

Staining was scored blinded to clinical data by two independent observers. ZFAND4 expression was evaluated according to the percentage of stained tumor cells and the staining intensity using the Allred score, as previously described [8]. The proportion of ZFAND4-positive cells was evaluated in both undifferentiated and differentiated areas of the tumors. In all these groups, proportional scores were categorized as: 0, no cells were stained; 1, 1/100 cells were stained; 2, 1/10 cells were stained; 4, 2/3 cells were stained; 5, all cells were stained. Staining intensity was scored as: 0, negative; 1, weak; 2, intermediate; and 3, strong. The total score was calculated by the sum of the proportional and intensity scores, ranging from 0 to 8. Similar to Kurihara-Shimomura et al. [6], the optimal cut-off score for ZFAND4 expression was selected using the receiver operating characteristic (ROC) curve according to the survival status. ZFAND4 staining was independently evaluated in undifferentiated areas of tumors mainly located at the invasive front, and in differentiated areas at the center of the tumor islands.

### 2.4. Statistical Analysis

χ^2^ and the Fisher’s exact test were used for comparison between categorical variables. Disease-specific survival (DSS) was determined for the date of treatment completion to death for the tumor. For time-to-event analysis, survival curves were plotted using the Kaplan-Meier method. Differences between survival times were analyzed by the log-rank test. Hazard ratios (HR) with their 95% confidence intervals (CI) for clinicopathologic variables were calculated using the univariate Cox proportional hazards model analysis. All tests were two-sided and *p* values less than 0.05 were considered statistically significant. All statistical analyses were performed using SPSS version 21 (IBM Co., Armonk, NY, USA).

## 3. Results

### 3.1. Patient Characteristics

The cohort of 125 OSCC patients was composed of 82 men and 43 women, ranging from 28 to 91 years, with a median age of 57 years. Forty-one patients (33%) were never-smokers and 56 (45%) never-drinkers. The main clinicopathologic characteristics are summarized in Table 1. Forty-nine cases (39%) showed neck lymph node metastasis, more than 50% were well-differentiated tumors and advanced clinical stages (III or IV), and the most common site was the tongue (41%) followed by the floor of the mouth (30%). Adjuvant radiotherapy was administered to 75 patients (60%), and adjuvant chemotherapy to 14 patients (11.2%). Fifty-four cases (43%) showed loco-regional recurrence, and 19 (15%) suffered from a second primary carcinoma. Over a median follow-up of 61 months (range, 1 to 230 months) 53 deaths occurred.

### 3.2. Immunohistochemical Analysis of ZFAND4 Expression in OSCC Tissue Specimens

**Figure 1:**
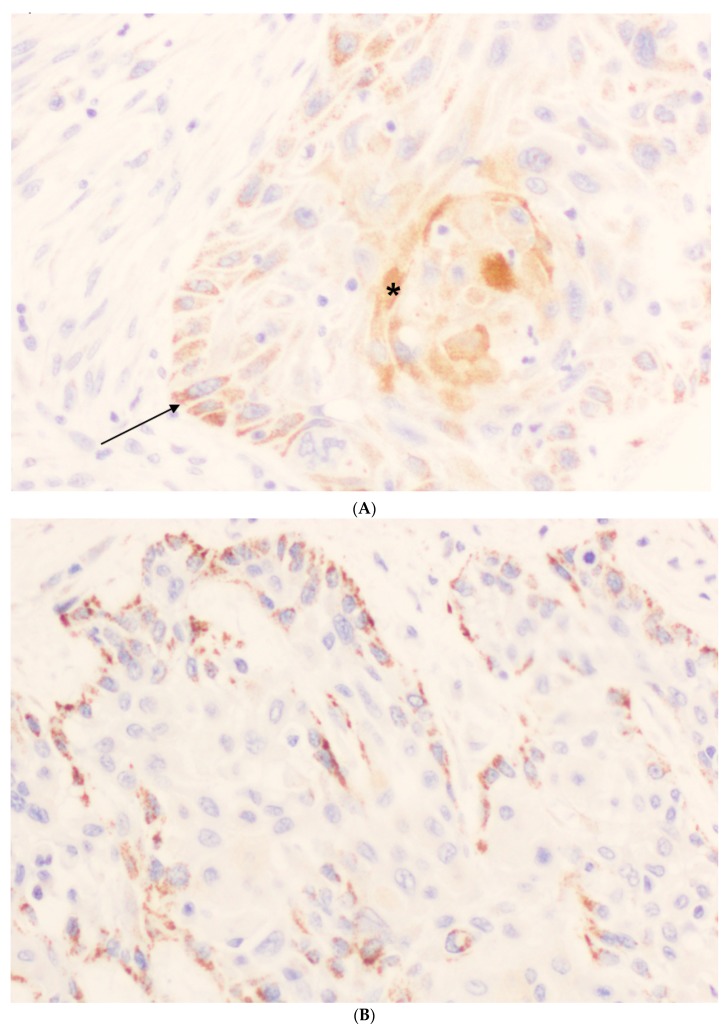

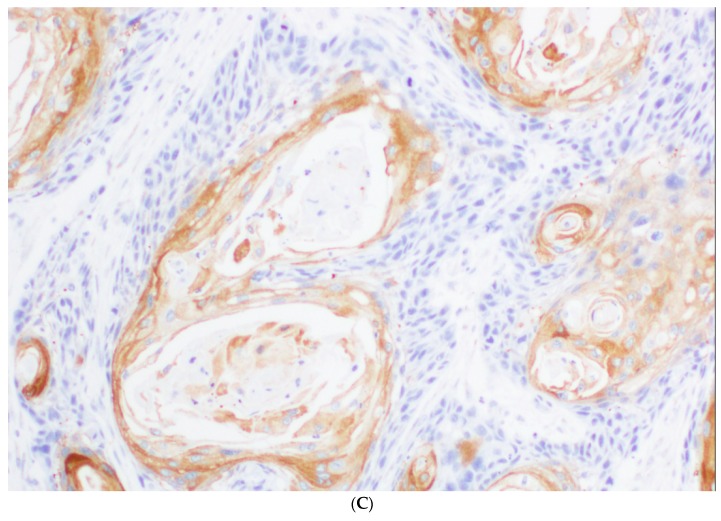
Immunoexpression of ZFAND4 in oral squamous cell carcinoma. (**A**) Staining in undifferentiated areas (arrow) and differentiated areas (*). (**B**) Staining in undifferentiated cells, mainly located in the invasive front of tumor tissue. (**C**) Staining in differentiated cells located at the center of the tumor islands.

ZFAND4 staining was not valuable in two (1.6%) of 125 OSCC specimens. While ZFAND4 expression was negligible in normal epithelium, two distinct expression patterns were noted in the tumors that did not overlap in any of the samples (Figure 1). A gross granular staining was characteristic of the undifferentiated cells at the invasive front of tumors, whereas the most differentiated cells located at the center of the tumor nests showed diffuse non-granular staining. The mean percentages of positive ZFAND4 staining were 44.98 (standard deviation –SD-35.38) in undifferentiated cells and 17.18 (SD, 20.61) in differentiated cells. ZFAND4 staining intensity was also evaluated in both undifferentiated and differentiated areas. In undifferentiated cells, there were 13 (11%) negative cases, 37 (30%) weak, 43 (35%) intermediate, and 30 (24%) cases with strong staining. In differentiated cells, 47 (38%) cases were scored negative, 11 (9%) weak, 47 (38%) intermediate, and 18 (15%) had strong staining. Since each tumor was represented by three different tissue cores in the OSCC TMAs, the percentages of stained cells frequently varied in the three tumor areas assessed. Taking this into consideration, the Allred score was determined in two different ways: Either considering the maximum value of ZFAND4 positivity or the mean value of the three tumor cores. Regarding the intensity of immunostaining, the maximum value was always used for all calculations. Finally, the total score was calculated by the sum of the percentages of staining and intensity scores. The resulting indexes ranged between 0 and 8. The receiver operating characteristics (ROC) curve was used to determine the best cut-off score to predict patients’ survival, and this value was 4. Accordingly, those cases with an Allred score above 4 were considered as high ZFAND4 expression.

### 3.3. Associations of ZFAND4 with Clinicopathologic Characteristics

We next assessed the correlations of high ZFAND4 expression with the clinical data. Table 2 shows the associations of high ZFAND4 expression determined by using the maximum value of the percentage of stained cells to calculate the Allred score. In differentiated areas, high ZFAND4 expression was significantly associated with well-differentiated (*p* = 0.04) and non-recurrent tumors (*p* = 0.04), whereas ZFAND4 expression in undifferentiated cells was significantly correlated with tumor location in the tongue (*p* = 0.005).

On the other hand, when the Allred score was calculated using the mean value of percentage of stained cells (Table 3), ZFAND4 expression in differentiated cells was found to be significantly associated with N status (*p* = 0.02), being more frequently detected in pN0 and pN1 cases compared to pN2. However, no significant relationship was found between ZFAND4 expression in undifferentiated cells and any clinicopathologic variable.

### 3.4. ZFAND4 Expression and Patients’ Survival

Over a median follow-up period of 61 months, 27 patients (42.1%) harboring high ZFAND4 expression in undifferentiated cells calculated by using the mean Allred score died due to the index cancer, and 15 patients (41.6%) with high ZFAND4 expression in the differentiated cells. When the maximum Allred score was used, 31 patients (40.7%) with high ZFAND4 expression in the undifferentiated cells, and 20 patients (36.3%) with high ZFAND4 expression in the differentiated cells died due to the index cancer. Kaplan-Meier analysis showed that there were no statistically significant differences in disease-specific survival (DSS) between patients with high versus low ZFAND4 expression in either differentiated or undifferentiated cells (Table 4).

## 4. Discussion

This study aimed to investigate the clinical relevance and prognostic significance of ZFAND4 in OSCC. The prevalence of OSCC is estimated at 264,000 cases and 128,000 deaths annually worldwide [3]. Since the completion of the Human Genome Project in 2003, the subsequent progress in understanding the biology of cancer has led to the development of personalized therapies based on the patient’s unique molecular and genetic profile to target defective signaling pathways of tumor cells. It is generally accepted that OSCC arises from multiple genetic alterations, although the molecular basis of carcinogenesis is not fully understood. DNA sequencing technologies coupled with advances in algorithms have enormously contributed to the molecular and functional characterization of mutations, genes, and pathways altered in multiple cancers, including OSCC [9,10,11,12]. Furthermore, the majority of tumors showed alterations in multiple targetable genes that are candidates for combination therapy [11]. It is of paramount importance to identify molecular alterations involved in the development of recurrent and metastatic disease, which remains the main cause of morbidity and mortality in OSCC patients.

In a recent paper, Sasahira et al. [5] conducted a cDNA microarray analysis in order to compare the gene expression profile of primary and recurrent OSCC. Ten genes were found to be upregulated in recurrent OSCC compared with the primary tumors. Among these genes, ZFAND4 showed a 100-fold recurrent/primary, thus suggesting a possible role for this gene in tumorigenesis. Tang et al. [7] reported that ZFAND4 expression was consistently highly expressed in gastric cancer compared to normal tissue, and positively associated with increased stage. Functionally, ZFAND4 was found to downregulate the expression of the anti-proliferative miRNAs, miR-148b, miR-375, and miR-182, in SGC-7901 cells, thereby promoting cell proliferation by activation of cyclin-dependent kinase and downregulation of p21 and p53 [7], which supports the notion that ZFAND4 may act as an oncogene in gastric cancer.

In this study, we assumed the same methodology used by Kurihara-Shimomura et al. [6] in order to validate their results in an independent cohort of OSCC patients, and more importantly, the utility of ZFAND4 as a predictor of metastasis and poor prognostic marker. Interestingly, ZFAND4 staining consistently showed distinct expression patterns and distribution in our cohort of 125 OSCC, i.e., granular staining in undifferentiated areas and diffuse staining in differentiated areas of the tumors. These two expression patterns were analyzed separately to evaluate possible correlations with the clinical and follow-up data. Moreover, Allred scores were calculated using both maximum value and mean value of ZFAND4-positive cells for the three tissue cores selected from each tumor.

We found that ZFAND4 staining was higher in undifferentiated than in differentiated areas of tumors. However, ZFAND4 expression in differentiated cells showed the most relevant and significant associations with well-differentiated (*p* = 0.04) and non-recurrent tumors (*p* = 0.04). Nevertheless, ZFAND4 expression did not show a major impact on patient survival. Only a trend was observed between low ZFAND4 expression in differentiated cells and tumor-associated deaths (Table 4). Therefore, the prognostic relevance of ZFAND4 described by Kurihara-Shimomura et al. [6] has not been replicated in our series. Furthermore, Kurihara-Shimomura et al. [6] reported that ZFAND4 is essential for distant metastasis in OSCC, and hypothesized that ZFAND4 could facilitate metastasis to the lymph nodes and distant organs by promoting angiogenesis and/or lymphangiogenesis. In marked contrast, our results only proved a marginal (if any) relationship between ZFAND4 expression and the presence of lymph node metastasis. The limitations of our study are the retrospective design and the use of tissue microarrays to evaluate ZFAND4 immunostaining. Several factors could contribute to the discrepant results between these two studies. On one hand, etiological, clinical, and epidemiological differences in the patient cohorts as well as molecular/biological differences among tumors depending on the geographic areas. Moreover, ZFAND4 expression exhibited a highly heterogeneous pattern within the tumors depending on the differentiation status, which could certainly have a major contribution on the varying results. Particularly since we evaluated separately the clinical significance and correlations of the two distinct ZFAND4 expression patterns in undifferentiated and differentiated areas of tumors, while Kurihara-Shimomura et al. [6] did not distinguish expression patterns within the different tumor areas. Thus, positive ZFAND4 immunostaining was scored in the whole tumor as the diffuse staining in tumor islands, cords, or sheets, irrespective of the cell differentiation status and the grade of keratinization. Since ZFAND4 expression showed two distinct non-overlapping expression patterns depending on the differentiation areas of tumors, each with a clearly distinct clinical significance, evaluation of ZFAND4 expression in the whole tumor could therefore be misleading.

## 5. Conclusions

The herein presented data question the clinical relevance of ZFAND4 expression as a prognostic biomarker in OSCC, and also revealed distinct ZFAND4 expression patterns depending on the differentiation areas of tumors that should be evaluated separately. Further studies are necessary to fully elucidate the pathobiological role of ZFAND4 in OSCC and the potential clinical implications.

## Figures and Tables

**Table 1:** Clinical and pathological characteristics of 123 patients with oral squamous cell carcinoma and where zinc finger AN1-type containing 4 (ZFAND4) was valuable.

Variable	Number (%)
Age (year) (mean ± SD; median; range)	58.6 ± 14.3; 57; 28–91
Gender	
Men	81 (66)
Women	42 (34)
Tobacco use	
Smoker	83 (67)
Non-smoker	40 (33)
Alcohol use	
Drinker	68 (55)
Non-drinker	55 (45)
Location of oral squamous oral cell carcinoma	
Tongue	51 (41)
Floor of the mouth	35 (28)
Other sites within te oral cavity	37 (31)
Tumor status	
pT1	27 (22)
pT2	53 (43)
pT3	15 (12)
pT4	28 (23)
Nodal status	
pN0	74 (60)
pN1	25 (20)
pN2	24 (20)
Clinical stage	
Stage I	20 (16)
Stage II	31 (25)
Stage III	25 (20)
Stage IV	47 (39)
G status	
G1	78 (63)
G2	41 (33)
G3	4 (4)
Second primary carcinoma	
No	104 (85)
Yes	19 (15)
Local recurrence	
No	69 (56)
Yes	54 (44)
Clinical status at the end of the follow-up	
Live and without recurrence	51 (41)
Dead of index cancer	53 (43)
Lost or died of other causes (censored)	19 (16)

**Table 2:** Relationships between clinical and pathological variables and high ZFAND4 expression determined by using the maximum value of stained cells to calculate the Allred score.

Variable	Number of Cases	High ZFAND4 Expression in Undifferentiated Cells (%)	*p*	High ZFAND4 Expression in Differentiated Cells (%)
Gender			0.98	
Men	81	50 (62)		38 (47)
Women	42	26 (62)		17 (40)
Tobacco use			0.77	
Smoker	83	52 (63)		34 (41)
Non-smoker	40	24 (60)		21 (52)
Alcohol use			0.99	
Drinker	68	42 (62)		31 (46)
Non-drinker	55	34 (62)		24 (44)
pT			0.44	
pT1	27	19 (70)		16 (59)
pT2	53	33 (62)		25 (47)
pT3	15	10 (67)		4 (27)
pT4	28	14 (50)		10 (36)
pN			0.75	
pN0	74	45 (61)		34 (46)
pN1	25	17 (68)		13 (52)
pN2	24	14 (58)		8 (33)
Clinical stage			0.69	
Stage I	20	13 (65)		9 (45)
Stage II	31	20 (64)		16 (52)
Stage III	25	17 (68)		11 (44)
Stage IV	47	26 (55)		19 (40)
G status			0.14	
G1 (Well)	78	52 (67)		40 (51)
G2 + G3 (Moderate + poor)	45	24 (53)		15 (33)
Tumor location			0.005	
Tongue	51	39 (76)		23 (45)
Rest	72	37 (51)		32 (44)
Tumor location			0.5	
Floor of the mouth	35	20 (57)		16 (46)
Rest	88	56 (64)		39 (44)
Tumor recurrence			0.61	
No	69	44 (64)		36 (52)
Yes	54	32 (59)		19 (35)
Second primary carcinoma			0.89	
No	104	64 (61)		48 (46)
Yes	19	12 (63)		7 (37)
Clinical status at the end of the follow-up			0.73	
Live and without recurrence	51	32 (63)		26 (51)
Dead of index cancer	53	31 (58)		20 (38)
Lost or died of other causes	19	13 (68)		9 (47)

**Table 3:** Relationships between clinical and pathological variables and high ZFAND4 expression determined by using the mean value of stained cells to calculate the Allred score.

Variable	Number of Cases	High ZFAND4 Expression in Undifferentiated Cells (%)	*p*	High ZFAND4 Expression in Differentiated Cells (%)	*p*
Gender			0.95		0.33
Men	81	42 (52)		26 (32)	
Women	42	22 (52)		10 (24)	
Tobacco use			0.75		0.58
Smoker	83	44 (53)		23 (28)	
Non-smoker	40	20 (50)		13 (32)	
Alcohol use			0.89		0.21
Drinker	68	35 (51)		23 (34)	
Non-drinker	55	29 (53)		13 (24)	
pT			0.29		0.33
pT1	27	16 (59)		11 (41)	
pT2	53	27 (51)		15 (28)	
pT3	15	10 (67)		2 (13)	
pT4	28	11 (39)		8 (29)	
pN			0.85		**0.02**
pN0	74	37 (50)		24 (32)	
pN1	25	14 (56)		10 (40)	
pN2	24	13 (54)		2 (8)	
Clinical stage			0.46		0.81
Stage I	20	10 (50)		6 (30)	
Stage II	31	17 (55)		11 (35)	
Stage III	25	16 (64)		9 (36)	
Stage IV	47	21 (45)		10 (21)	
G status			0.36		0.08
G1 (Well)	78	43 (55)		27 (35)	
G2 + G3 (Moderate + poor)	45	21 (47)		9 (20)	
Tumor location			0.1		0.43
Tongue	51	31 (61)		13 (25)	
Rest	72	33 (46)		23 (32)	
Tumor location			0.75		0.44
Floor of the mouth	35	19 (54)		12 (34)	
Rest	88	45 (51)		24 (27)	
Tumor recurrence			0.97		0.47
No	69	36 (52)		22 (32)	
Yes	54	28 (52)		14 (26)	
Second primary carcinoma			0.29		0.75
No	104	52 (50)		31 (30)	
Yes	19	12 (63)		5 (26)	
Clinical status at the end of the follow-up			0.85		0.59
Live and without recurrence	51	26 (51)		17 (33)	
Dead of index cancer	53	27 (51)		15 (28)	
Lost or died of other causes	19	11 (58)		4 (21)	

**Table 4:** Univariate Kaplan-Meier and Cox analysis to assess the association of ZFAND4 expression on disease-specific survival in oral squamous cell carcinoma patients.

ZFAND4 Expression	Censored Patients (%)	Mean Survival Time (95% CI)	HR (95% CI)	*p*
Undifferentiated cells				0.7
calculated by using the mean Allred score				
Low	33 (56)	107.72 (86.32–129.12)	Reference	
High	37 (58)	135.20 (108.87–161.52)	0.90 (0.52–1.55)	
Differentiated cells				0.8
calculated by using the mean Allred score				
Low	49 (56)	126.62 (104.38–148.85)	Reference	
High	21 (58)	135.11 (99.60–170.62)	0.93 (0.51–1.69)	
Undifferentiated cells				0.53
calculated by using the maximum Allred score				
Low	25 (53)	104.14 (80.46–127.82)	Reference	
High	45 (59)	136.44 (111.81–161.06)	0.84 (0.48–1.45)	
Differentiated cells				0.25
calculated by using the maximum Allred score				
Low	35 (51)	105.55 (85.11–126.00)	Reference	
High	35 (64)	144.08 (114.95–173.21)	0.72 (0.41–1.26)	
